# De novo* SCN8A* and inherited rare *CACNA1H* variants associated with severe developmental and epileptic encephalopathy

**DOI:** 10.1186/s13041-021-00838-y

**Published:** 2021-08-16

**Authors:** Robin N. Stringer, Bohumila Jurkovicova-Tarabova, Ivana A. Souza, Judy Ibrahim, Tomas Vacik, Waseem Mahmoud Fathalla, Jozef Hertecant, Gerald W. Zamponi, Lubica Lacinova, Norbert Weiss

**Affiliations:** 1 Department of Pathophysiology, Third Faculty of Medicine, Charles University, Prague, Czech Republic; 2 Institute of Organic Chemistry and Biochemistry, Czech Academy of Sciences, Prague, Czech Republic; 3 Center of Biosciences, Institute of Molecular Physiology and Genetics, Slovak Academy of Sciences, Bratislava, Slovakia; 4 Department of Physiology and Pharmacology, Cumming School of Medicine, University of Calgary, Calgary, Canada; 5 Department of Pediatrics, Tawam Hospital, Al-Ain, United Arab Emirates; 6 Institute of Biology and Medical Genetics, First Faculty of Medicine, Charles University, Prague, Czech Republic; 7 Department of Pediatric Neurology, Mafraq Hospital, Abu Dhabi, United Arab Emirates; 8 Department of Pediatrics, College of Medicine and Health Sciences, United Arab Emirates University, Al-Ain, United Arab Emirates

**Keywords:** Ion channels, Channelopathy, Calcium channel, *CACNA1H*, Ca_v_3.2 channel, Sodium channel, *SCN8A*, Na_v_1.6 channel, Epilepsy, Encephalopathy

## Abstract

Developmental and epileptic encephalopathies (DEEs) are a group of severe epilepsies that are characterized by seizures and developmental delay. DEEs are primarily attributed to genetic causes and an increasing number of cases have been correlated with variants in ion channel genes. In this study, we report a child with an early severe DEE. Whole exome sequencing showed a de novo heterozygous variant (c.4873–4881 duplication) in the *SCN8A* gene and an inherited heterozygous variant (c.952G > A) in the *CACNA1H* gene encoding for Na_v_1.6 voltage-gated sodium and Ca_v_3.2 voltage-gated calcium channels, respectively. In vitro functional analysis of human Na_v_1.6 and Ca_v_3.2 channel variants revealed mild but significant alterations of their gating properties that were in general consistent with a gain- and loss-of-channel function, respectively. Although additional studies will be required to confirm the actual pathogenic involvement of *SCN8A* and *CACNA1H*, these findings add to the notion that rare ion channel variants may contribute to the etiology of DEEs.

**Supplementary Information:**

The online version contains supplementary material available at 10.1186/s13041-021-00838-y.

## Main text

Developmental and epileptic encephalopathies (DEEs) are a group of severe epilepsies that are characterized by seizures often drug-resistant, and developmental delay leading to varying degrees of intellectual, psychiatric, behavioral, and motor disabilities [1]. DEEs are primarily attributed to genetic causes and while recessive and X-linked variants have been found, the majority of patients show de novo pathogenic variants [2]. Recently, an increasing number of DEE cases have been correlated with variants in ion channel genes [3].

**Fig. 1:**
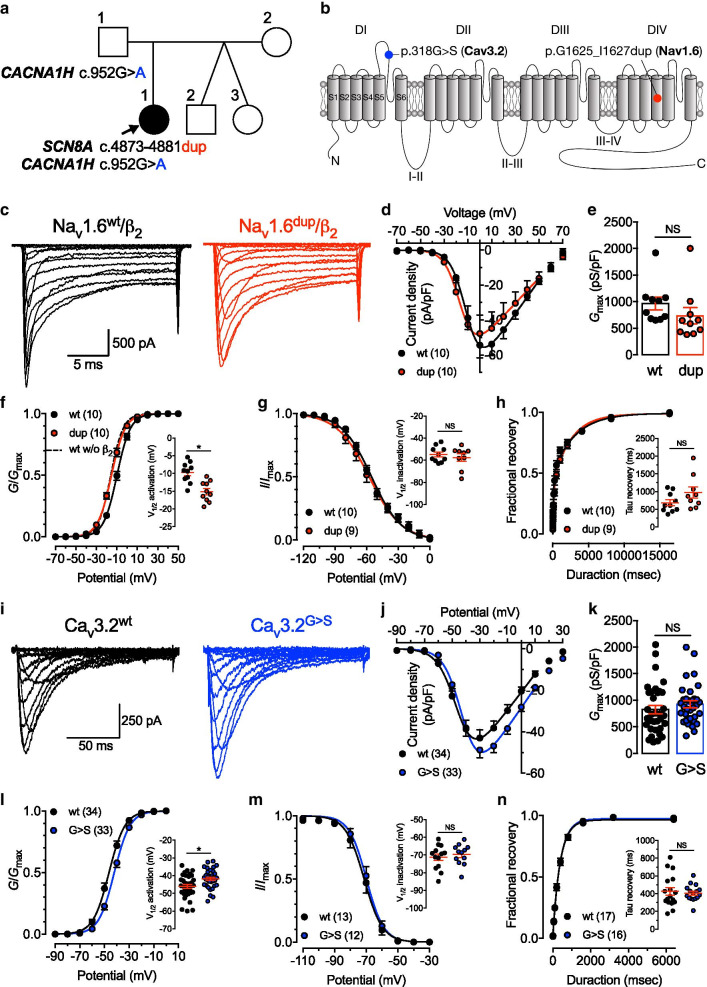
Electrophysiological properties of Na_v_1.6 and Ca_v_3.2 channel variants associated with developmental and epileptic encephalopathy. **a** Family pedigree chart. Filled and open symbols indicate affected and unaffected individuals, respectively. **b** Location of the Na_v_1.6 G1625_I1627 duplication (red circle) and Ca_v_3.2 G318S missense variants (blue circle) within the secondary membrane topology of the channels. **c** Representative sodium current traces recorded from cells expressing wild-type Na_v_1.6 (Na_v_1.6^wt^, black traces) and Na_v_1.6 duplication variant (Na_v_1.6^dup^, red traces) in combination with Na_v_b_2_. **d** Corresponding mean current–voltage (*I*/*V*) relationship. **e** Corresponding mean maximal macroscopic conductance (*G*_max_) values obtained from the fit of the *I*/*V* curves with the modified Boltzmann Eq. (1). **f** Corresponding mean normalized voltage-dependence of activation. The voltage-dependence of activation for Na_v_1.6^wt^ in the absence of Na_v_b_2_ is shown for comparison (dotted line). *Inset* shows corresponding mean half-activation potential values obtained from the fit of the activation curve with the modified Boltzmann Eq. (2). **g** Mean normalized voltage-dependence of steady-state inactivation for Na_v_1.6^wt^ and Nav1.6^dup^. *Inset* shows corresponding mean half-inactivation potential values obtained from the fit of the inactivation curves with the two-state Boltzmann function (3). **h** Mean normalized recovery from inactivation kinetics. *Inset* shows corresponding mean time constant t values of recovery from inactivation obtained by fitting recovery curves with a single-exponential function (4). **i-n** Legend same as in (c-h) but for cells expressing wild type Ca_v_3.2 (Ca_v_3.2^wt^, black) and Ca_v_3.2 G318S (Ca_v_3.2^G>S^, blue) channel variants

In the present study, we report a girl with an early severe DEE. She was born by emergency caesarean section at 37 weeks due to placenta previa and was the first child of non-consanguineous parents. Immediately after birth, she presented with trembling despite normal blood sugar levels. In the early postnatal period, she developed myoclonic jerks in all limbs, diagnosed as infantile spasms but did not respond to steroids. By the age of 2 months, she started having generalized tonic–clonic seizures and recurrent status epilepticus that poorly responded to antiepileptic medication including clobazam, levetiracetam, phenobarbital and topiramate. Seizures were characterized by right eye deviation and generalized tonic posturing. She also presented with additional complications including scoliosis, bilateral hip dislocation and recurrent pneumonia, and by the age of 3 she developed myoclonus, spastic quadriplegia with generalized hypertonia and hyperreflexia with clonus. Secondary skeletal abnormalities were also observed including flattening of the head and chest, severe kyphoscoliosis and flexion contractures. An MRI brain scan showed generalized brain atrophy with marked insular atrophy and bright white matter on flair. Blood tests were in general normal and only creatine phosphokinase levels were increased, probably as secondary consequence of seizures. The patient died at the age of 4. Whole exome sequencing (EGL Genetics) showed a de novo heterologous duplication (c.4873_4881dup) in *SCN8A* (Fig. 1a) causing the duplication of amino acid G1625_I1627 (p.G1625_I1627dup) within the highly conserved transmembrane IVS4 segment (voltage sensor) of the voltage-gated sodium channel Na_v_1.6 (Fig. 1b). This variant has never been reported in the Genome Aggregation Database (gnomAD) and was predicted to be deleterious (PROVEAN algorithm). In addition, a rare heterozygous missense variant (c.952G > A) in *CACNA1H* (Fig. 1a) was inherited from the father who was asymptomatic. This variant that caused the substitution of a glycine at position 318 by a serine (p.G318S) within the first pore-forming loop of the voltage-gated calcium channel Ca_v_3.2 (Fig. 1b) has never been reported and was not predicted to be deleterious. To assess the impact of these mutations, the G1625_I1627 duplication and G318S missense variant were introduced into the human Na_v_1.6 (UniProt Q9UQD0-1) and Ca_v_3.2 (UniProt O95180-1) channels, respectively, and recombinant channels were expressed in HEK cells for electrophysiological analysis. The sodium conductance recorded from cells expressing the duplication variant (Na_v_1.6^dup^) in combination with the human Na_v_b_2_ ancillary subunit (Uniprot O60939) was similar to the one measured from cells expressing the wild-type channel (Na_v_1.6^wt^) (Fig. 1c–e and Additional file 1: Table S1). However, the mean half activation potential of Na_v_1.6^dup^ was shifted toward more hyperpolarized potentials by − 5.4 mV (*p* = 0.0005) (Fig. 1f and Additional file 1: Table S1) to values similar to Na_v_1.6^wt^ expressed without the Na_v_b_2_ subunit (Additional file 1: Fig. S1 and Table S1). In contrast, we did not observe any gating alteration of Na_v_1.6^dup^ in the absence of Na_v_b_2_. While the current literature on the effect of Na_v_b on the regulation of Na_v_1.6 is rather sparse and conflicting [4, 5], these results suggest that phenotypic expression of *SCN8A* duplication variant may depend on the molecular composition of Na_v_1.6, possibly by disrupting Na_v_b-dependent regulation of the channel. Other gating properties including steady-state inactivation and recovery from inactivation were not affected (Fig. 1g, h and Additional file 1: Table S1). In addition, recording of T-type currents from cells expressing the Ca_v_3.2 G318S variant (Ca_v_3.2^G>S^) did not reveal any alteration of the T-type conductance compared to cells expression the wild-type channel (Ca_v_3.2^wt^) (Fig. 1i–k and Additional file 1: Table S1). However, the mean half activation potential of the Ca_v_3.2^G>S^ variant was shifted toward more positive potentials by + 4.3 mV (*p* = 0.0048) (Fig. 1l and Additional file 1: Table S1) without any additional alteration of the other gating properties (Fig. 1m, n and Additional file 1: Table S1).

In summary, we reported the case of a child with severe DEE in whom a de novo mutation in *SCN8A* and an inherited rare *CACNA1H* variant were found. Pathogenic variants in *SCN8A* have originally been described in patients with DEE [6–9]. Most are de novo missense variants clustered in the highly conserved transmembrane domains of Na_v_1.6 and are in general consistent with a gain-of-function pathogenic mechanism predicted to increase neuronal excitability and seizure susceptibility [6, 10, 11]. Our observation that the *SCN8A* duplication variant produced a hyperpolarizing shift of the voltage-dependence of activation of Na_v_1.6 is also consistent with a gain-of-function (GoF) of the channel. Although future studies will be required to further assess the importance of the molecular composition of the channel in the phenotypic expression of *SCN8A* variants, the results presented here strengthen the notion that GoF *SCN8A* mutations may represent a general pathogenic mechanism in DEEs. In contrast, *CACNA1H* has never been associated with DEEs. Instead, GoF *CACNA1H* variants have been linked to absence epilepsy and primary aldosteronism [12] while loss-of-function (LoF) variants have been reported in autism spectrum disorders [13], amyotrophic lateral sclerosis [14, 15], and congenital amyotrophy [16]. It is not clear to which extent the LoF *CACNA1H* variant we identified in our patient may have contributed to the disease. Given that the father from whom the child inherited this variant was asymptomatic, this variant may not have had a major contribution to the development of the disease on its own. However, it is a possibility that it may have precipitated its development by interacting with other genes. This notion is supported by previous studies showing that *CACNA1G* (Ca_v_3.1) and *CACNA1A* (Ca_v_2.1) are genetic modifiers of epilepsy associated with Dravet syndrome [17–19]. While additional studies using primary neurons will be required to uncover the detailed underlying pathogenic mechanisms of Na_v_1.6 and Ca_v_3.2 variants, the current findings add to the notion that rare ion channel variants may contribute to the etiology of DEEs.

## Supplementary Information

**Additional file 1: Fig. S1.** Electrophysiological properties of Nav1.6 variant expressed in the absence of Navb2. a Representative sodium current traces recorded from cells expressing wild-type Nav1.6 (Nav1.6wt, black traces) and Nav1.6 duplication variant (Nav1.6dup, red traces). b Corresponding mean current–voltage (I/V) relationship. c Corresponding mean maximal macroscopic conductance (Gmax) values obtained from the fit of the I/V curves with the modified Boltzmann Eq. (1). d Corresponding mean normalized voltage dependence of activation. Inset shows corresponding mean half-activation potential values obtained from the fit of the activation curve with the modified Boltzmann Eq. (2). e Mean normalized voltage-dependence of steady-state inactivation for Nav1.6wt and Nav1.6dup. Inset shows corresponding mean half-inactivation potential values obtained from the fit of the inactivation curves with the two-state Boltzmann function (3). f Mean normalized recovery from inactivation kinetics. Inset shows corresponding mean time constant t values of recovery from inactivation obtained by fitting recovery curves with a single-exponential function (4). **Table S1.** Electrophysiological properties of human Nav1.6 and Cav3.2 variants expressed in tsA-201 cells. *p < 0.05.

## Data Availability

All data generated or analyzed during this study are included in this published article and its additional information files.

## Abbreviations

DEEs: Developmental and epileptic encephalopathies
GoF: Gain-of-function
LoF: Loss-of-function
