# Supplementary material for: De novo SCN8A and inherited rare CACNA1H variants associated with severe developmental and epileptic encephalopathy
Source: Mol Brain. 2021 Aug 16;14:126. doi: 10.1186/s13041-021-00838-y (PMC8365958; doi:10.1186/s13041-021-00838-y)
Supplement: Supplementary file 1 — Additional file 1: Fig. S1. Electrophysiological properties of Nav1.6 variant expressed in the absence of Navb2. a Representative sodium current traces recorded from cells expressing wild-type Nav1.6 (Nav1.6wt, black traces) and Nav1.6 duplication variant (Nav1.6dup, red traces). b Corresponding mean current–voltage (I/V) relationship. c Corresponding mean maximal macroscopic conductance (Gmax) values obtained from the fit of the I/V curves with the modified Boltzmann Eq. (1). d Corresponding mean normalized voltage dependence of activation. Inset shows corresponding mean half-activation potential values obtained from the fit of the activation curve with the modified Boltzmann Eq. (2). e Mean normalized voltage-dependence of steady-state inactivation for Nav1.6wt and Nav1.6dup. Inset shows corresponding mean half-inactivation potential values obtained from the fit of the inactivation curves with the two-state Boltzmann function (3). f Mean normalized recovery from inactivation kinetics. Inset shows corresponding mean time constant t values of recovery from inactivation obtained by fitting recovery curves with a single-exponential function (4). Table S1. Electrophysiological properties of human Nav1.6 and Cav3.2 variants expressed in tsA-201 cells. *p < 0.05. [file 13041_2021_838_MOESM1_ESM.pdf]

De novo SCN8A and inherited rare _CACNA1H_ variants associated with severe developmental and epileptic encephalopathy

Additional information

Additional methods

Site-directed mutagenesis

The $Na_v1.6$ G1625_I1627 duplication variant cDNA construct was generated by introducing the mutation into the human $Na_v1.6$ (UniProt Q9UQD0-1, obtained from anaxon®) in pcDNA5FRT by PCR using Q5 Site-Directed Mutagenesis Kit (NEB) and the following mutagenic primers: forward: 5´-GATCCTGCGGCTGATCAAGGGCG-3´; reverse: 5´-CTTCCGATTCTGCCGATCCGGGCC-3´. The $Ca_v3.2$ G318S variant was generated using the human $Ca_v3.2$ (UniProt O95180-1) in pcDNA3 and the following primers: forward: 5'-CCTGCACCCTGAGCTGGGAGGCC-3'; reverse: 5'-GGCCTCCCAGCTCAGGGTGCAGG-3'. Final constructs were verified by sequencing of the coding region of the plasmid cDNAs.

Cell culture and heterologous expression

Human embryonic kidney HEK-293T and tsA-201 cells were grown in DMEM medium supplemented with 10% fetal bovine serum and 1% penicillin/streptomycin (all media purchased from Invitrogen) and maintained under standard conditions at 37^oC in a humidified atmosphere containing 5% CO_2. Heterologous expression of $Na_v1.6$ (in HEK-293T cells) and $Ca_v3.2$ channels (in tsA-201 cells) was performed by transfecting cells with plasmid cDNAs using the calcium/phosphate and jetPRIME transfection reagent, respectively. Empty pEGFP vector was used as transfection marker.

Patch clamp recordings

Patch clamp recordings were conducted at room temperature (22-25^0C) in the whole-cell configuration using a HEKA-10 (HEKA Electronic) or Axopatch 200B (Axon Instruments)

amplifier to assess $Na_v1.6$ and $Ca_v3.2$ channels, respectively. Pipette resistance was between 1.5 and 2.5 MΩ and series resistance was between 2.5 to 5 MΩ, and was compensated up to 70%. The linear capacity and leakage currents were subtracted using the P/4 procedure. For recording of sodium currents, the extracellular solution contained (in mM): 105 NaCl, 2 CaCl2, 0.5 MgCl2, 10 4-(2-hydroxyethyl)-1-piperazineethanesulfonic acid (HEPES), 25 TEA-Cl, and 10 glucose, pH 7.4 titrated with NaOH. The pipette solution contained (in mM): 135 CsCl, 3 EGTA, 2 MgCl2, 20 TEA-Cl, 5 Na2-ATP, and 10 HEPES, pH 7.4 titrated with CsOH. Recordings were performed from a holding potential (HP) of −100 mV. Current/voltage (I/V) relationships of sodium inward currents were measured by a series of 20 ms long depolarizing pulses applied from HP to membrane potentials ranging from −70 to +80 mV with 10 mV increment. The voltage-dependence of steady-state inactivation was measured by a series of 1000 ms long conditioning prepulses to potentials ranging from −120 to 0 mV with 10 mV increment, followed by 5 ms long test pulses to 0 mV. Recovery from inactivation was assessed using a 5 ms conditioning pulse to 0 mV from a holding potential of −100 mV, followed by a 10 s long prepulse to 0 mV and a recovery interval of variable duration (Δ2 ms) and a 5 ms test pulse to 0 mV. For recording of T-type currents, the extracellular solution contained (in millimolar): 5 $BaCl_2$, 5 KCl, 1 $MgCl_2$, 128 NaCl, 10 TEA-Cl, 10 D-glucose, 10 HEPES, pH 7.2 titred with NaOH. The pipette solution contained (in millimolar): 110 CsCl, 3 Mg-ATP, 0.5 Na-GTP, 2.5 $MgCl_2$, 5 D-glucose, 10 EGTA, and 10 HEPES, pH 7.4 titred with CsOH. Current/voltage (I/V) relationships of T-type currents was determined by measuring the peak T-type current amplitude in response to 150 ms depolarizing steps to various potentials applied every 10 s from a holding membrane potential of -100 mV. The voltage-dependence of the steady-state inactivation of $Ca_v3.2$ channels was determined by measuring the peak T-type current amplitude in response to a 150 ms depolarizing step to -20 mV applied after a 5 s-long conditioning prepulse ranging from -120 mV to -30 mV. The current amplitude obtained during each test pulse was normalized to the maximal current amplitude and plotted as a function of the prepulse potential. The recovery from inactivation was assessed using a double-pulse protocol from a holding potential of -100 mV. The cell membrane was depolarized for 2 s at 0 mV (inactivating prepulse) to ensure complete inactivation of the channel, and then to -20 mV for 150 ms

(test pulse) after an increasing time period (interpulse) ranging between 0.1 ms and 7 s at -100 mV. The peak current from the test pulse was plotted as a ratio of the maximum prepulse current versus interpulse interval.

Mathematical analysis

The current-voltage relationship (I/V) curve was fitted with the following modified Boltzmann equation (1):

$$(1) \qquad I(V) = Gmax \frac{(V - V\mathrm{rev})}{1 + \exp\frac{(V0.5 - V)}{k}}$$

with $I(V)$ being the peak current amplitude at the command potential V, G_{max} the maximum conductance, V_{rev} the reversal potential, $V_{0.5}$ the half-activation potential, and k the slope factor. The voltage-dependence of the whole-cell conductance was calculated using the following modified Boltzmann equation (2):

$$(2) \qquad G(V) = \frac{Gmax}{1 + \exp\frac{(V0.5 - V)}{k}}$$

with $G(V)$ being the conductance at the command potential V. The voltage-dependence of the steady-state inactivation was fitted with the following two-state Boltzmann function (3):

$$(3) \qquad I(V) = \frac{Imax}{1 + \exp\frac{(V - V0.5)}{k}}$$

with I_{max} corresponding to the maximal peak current amplitude and $V_{0.5}$ to the half-inactivation voltage. The recovery from inactivation curve was fitted with the following single-exponential function (4):

$$(4) \qquad \frac{I}{Imax} = A \times (1 - exp\frac{-t}{\tau})$$

where τ is the time constant for channel recovery from inactivation.

Statistical analysis

Data values are presented as mean ± S.E.M for *n* measurements. Statistical analysis was performed using GraphPad Prism 7. For datasets passing the D'Agostino & Person omnibus normality test, statistical significance was determine using a Student's t-test. Datasets were considered significantly different for $p \leq 0.05$ *.

Additional figures and Tables

Additional Figure S1

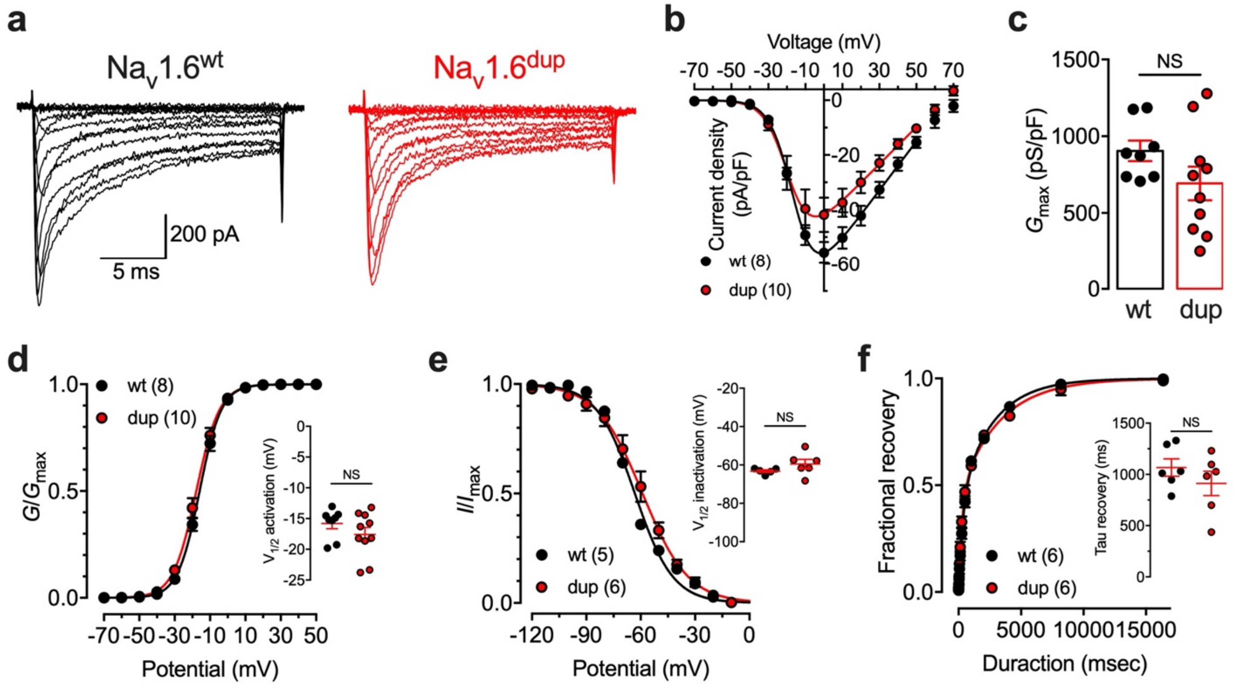

Fig.S1 Electrophysiological properties of Na$_v$1.6 variant expressed in the absence of Na$_v\beta_2$. **a** Representative sodium current traces recorded from cells expressing wild-type Na$_v$1.6 (Na$_v$1.6^{wt}, black traces) and Na$_v$1.6 duplication variant (Na$_v$1.6^{dup}, red traces). **b** Corresponding mean current-voltage (*I*/*V*) relationship. **c** Corresponding mean maximal macroscopic conductance (G_{max}) values obtained from the fit of the *I*/*V* curves with the modified Boltzmann equation (1). **d** Corresponding mean normalized voltage-dependence of activation. *Inset* shows corresponding mean half-activation potential values obtained from the fit of the activation curve with the modified Boltzmann equation (2). **e** Mean normalized voltage-dependence of steady-state inactivation for Na$_v$1.6^{wt} and Nav1.6^{dup}. *Inset* shows corresponding mean half-inactivation potential values obtained from the fit of the inactivation curves with the two-state Boltzmann function (3). **f** Mean normalized recovery from inactivation kinetics. *Inset* shows corresponding mean time constant τ values of recovery from inactivation obtained by fitting recovery curves with a single-exponential function (4).

Additional Table S1. Electrophysiological properties of human $Na_v1.6$ and $Ca_v3.2$ variants expressed in tsA-201 cells. $^*p < 0.05$.

	Activation				Inactivation			RFI	
**Channel variant**	**$V_{0.5}$ (mV)**	***k***	**G_{max} (pS/pF)**	**(n)**	**$V_{0.5}$ (mV)**	***k***	**(n)**	**τ (ms)**	**(n)**
**$Na_v1.6^{wt}$**	-15.8 ± 0.9	6.0 ± 0.3	905 ± 67	8	-63.2 ± 0.7	-10.5 ± 0.3	5	1066 ± 85	6
**$Na_v1.6^{dup}$**	-17.6 ± 1.2	6.2 ± 0.2	693 ± 109	10	-59.5 ± 2.4	-12.2 ± 1.5	6	912 ± 119	6
**$Na_v1.6^{wt}/\beta_2$**	-9.7 ± 0.9	6.4 ± 0.2	965 ± 120	10	-54.7 ± 2.2	-14.2 ± 1.0	10	686 ± 85	10
**$Na_v1.6^{dup}/\beta_2$**	**-15.1 ± 0.9***	6.5 ± 0.2	735 ± 154	10	-57.5 ± 3.3	-14.3 ± 0.7	9	972 ± 157	9
**$Ca_v3.2^{wt}$**	-46.1 ± 1.1	5.0 ± 0.2	825 ± 79	34	-71.2 ± 1.9	-4.1 ± 0.2	13	427 ± 44	17
**$Ca_v3.2^{G>S}$**	**-41.8 ± 1.0***	5.4 ± 0.2	924 ± 66	33	-69.7 ± 1.7	-4.2 ± 0.3	12	406 ± 23	16
